# Why Be One Protein When You Can Affect Many? The Multiple Roles of YB-1 in Lung Cancer and Mesothelioma

**DOI:** 10.3389/fcell.2019.00221

**Published:** 2019-10-01

**Authors:** Thomas G. Johnson, Karin Schelch, Sunali Mehta, Andrew Burgess, Glen Reid

**Affiliations:** ^1^Asbestos Diseases Research Institute, Sydney, NSW, Australia; ^2^Cell Division Laboratory, The ANZAC Research Institute, Sydney, NSW, Australia; ^3^School of Medicine, The University of Sydney, Sydney, NSW, Australia; ^4^Sydney Catalyst Translational Cancer Research Centre, The University of Sydney, Sydney, NSW, Australia; ^5^Institute of Cancer Research, Medical University of Vienna, Vienna, Austria; ^6^Department of Pathology, University of Otago, Dunedin, New Zealand; ^7^Maurice Wilkins Centre, University of Otago, Dunedin, New Zealand

**Keywords:** lung cancer, mesothelioma, targeted therapy, biomarker, Y-box binding protein-1

## Abstract

Lung cancers and malignant pleural mesothelioma (MPM) have some of the worst 5-year survival rates of all cancer types, primarily due to a lack of effective treatment options for most patients. Targeted therapies have shown some promise in thoracic cancers, although efficacy is limited only to patients harboring specific mutations or target expression. Although a number of actionable mutations have now been identified, a large population of thoracic cancer patients have no therapeutic options outside of first-line chemotherapy. It is therefore crucial to identify alternative targets that might lead to the development of new ways of treating patients diagnosed with these diseases. The multifunctional oncoprotein Y-box binding protein-1 (YB-1) could serve as one such target. Recent studies also link this protein to many inherent behaviors of thoracic cancer cells such as proliferation, invasion, metastasis and involvement in cancer stem-like cells. Here, we review the regulation of YB-1 at the transcriptional, translational, post-translational and sub-cellular levels in thoracic cancer and discuss its potential use as a biomarker and therapeutic target.

## Introduction

Lung cancers are the leading cause of cancer death worldwide (Islami et al., 2015; Kris et al., 2017), and malignant pleural mesothelioma patients continue to experience some of the worst 5-year survival rates of all malignancies (Mutti et al., 2018). Therefore, advances in therapeutic options are urgently needed and require a more thorough understanding of the underlying biology of both.

While SCLC represents ∼15–20% of all lung cancers, NSCLC represent the majority of cases (∼80–85%). NSCLC are further subtyped into adenocarcinomas (ADC; ∼40–50% of NSCLC), squamous cell carcinomas (SCC; ∼20–40%) and large cell carcinomas (LGC; ∼20%). Whilst all of these carcinomas are significantly associated with tobacco consumption, this association is much stronger in SCLC and SCC than in ADC and LGC (Khuder, 2001).

Malignant pleural mesothelioma arises from the pleural linings of the lung and is strongly linked to asbestos exposure (Tossavainen, 2004). MPM is currently subtyped as epithelioid, sarcomatoid or biphasic, which are characterized by a mixture of epithelioid and sarcomatoid cells (Marshall et al., 2015). At times, this review refers to lung cancer and mesothelioma as “thoracic cancers,” although we acknowledge that this term also encompasses tumors of the trachea, esophagus and thymus.

The current clinical practice guidelines for NSCLC, SCLC, and MPM all recommend the use of platinum-based chemotherapy in combination with other agents as the standard mode of care (Vogelzang et al., 2003; Rudin et al., 2016; Bradbury et al., 2017; Kris et al., 2017; Nagasaka and Gadgeel, 2018; Szolkowska et al., 2018). Diagnosis in the early stages of NSCLC affords better survival odds, however, the majority of patients are diagnosed with advanced disease (Kris et al., 2017; Visconti et al., 2017). Such individuals face a 5-year survival rate of only 23% and treatment options are often limited to chemotherapy (Kris et al., 2017). SCLC patients face similarly poor survival odds. Patients usually respond initially to platinum-based chemotherapy but inevitably develop chemoresistant tumors (Rudin et al., 2016). Overall survival rates of SCLC patients currently sit at 10–12 months post diagnosis (Rudin et al., 2016). In MPM, the standard of care consists of a combination of cisplatin with pemetrexed, providing an overall survival rate of only 12.1 months (Vogelzang et al., 2003; Mutti et al., 2018). Epithelioid mesotheliomas present with the best prognosis, with the median overall survival being between 12 and 27 months (Yap et al., 2017). Patients with biphasic mesothelioma have median overall survival rates of 7–18 months, while sarcomatoid patients are afforded the worst prognosis of 4–12 months (Yap et al., 2017). Recent trials of immunotherapy strategies, such as the anti-PD-1 checkpoint inhibitors pembrolizumab and nivolumab, have shown promise as first-line and second-line therapies in some thoracic cancers (Visconti et al., 2017; Forde et al., 2019). However, response to immunotherapy is unpredictable due to a lack of robust biomarkers, so predicating which patients will respond is not yet possible (Ventola, 2017). Acquired resistance to these drugs also remains a significant problem (Ventola, 2017). Improved treatment options for patients suffering malignancies of the lung and mesothelial linings are therefore still desperately needed.

### Toward Personalized Therapy for Thoracic Cancer Patients

The development of next-generation sequencing has fostered a deeper understanding of the molecular drivers and mutational landscape of thoracic cancers. Multi-region whole-exome sequencing of 100 early stage NSCLC patients demonstrated that clonal alterations of oncogenes such as the growth receptor *EGFR* and the kinases *MET*, and *BRAF* were commonly found in ADC (Jamal-Hanjani et al., 2017). These were accompanied by sub-clonal modifications of the oncogene *PIK3CA* and the tumor suppressor neurofibromin 1 (Jamal-Hanjani et al., 2017). Alterations of *PIK3CA*, the transmembrane receptor *NOTCH1*, growth factor receptor *FGFR1* and transcription factor *SOX2* were also observed in early SCC (Jamal-Hanjani et al., 2017). *TP53* or p53 mutations were frequent clonal events in both subtypes, while oncogenic *ALK* translocations were not observed in any tumors (Jamal-Hanjani et al., 2017).

**FIGURE 1:**
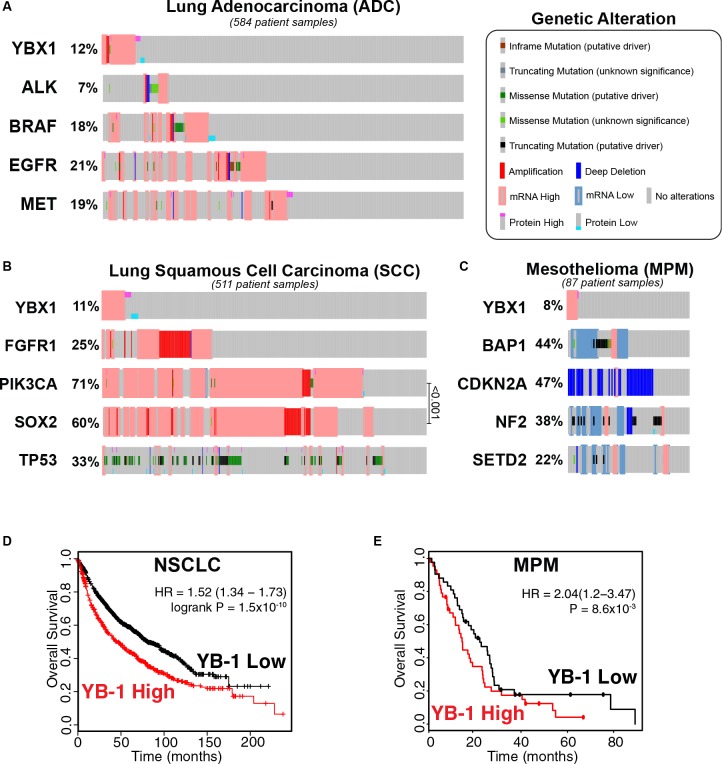
YB-1 is altered in NSCLC (ADC and SCC) and MPM patients and high *YBX1* mRNA expression correlates with poor prognosis in both diseases. Reported alteration frequencies of *YBX1* and commonly altered genes in current TCGA Provisional datasets for all complete tumors with RNASeq V2 RSEM mRNA and RPPA protein Expression for **(A)** Lung Adenocarcinoma (ADC; *n* = 584), **(B)** Lung Squamous Cell Carcinoma (SCC; *n* = 511) and **(C)** Mesothelioma (MPM; *n* = 87). Panels **(A–C)** were adapted from the open-source platform cBioPortal for Cancer Genomics (cBioPortal.org). **(D)** High *YBX1* expression correlates with poor prognosis in NSCLC patients (*p* = 1.5 × 10^–10^). Kaplan-Meir plot of 1,926 NSCLC patients generated using Lung Cancer KM plotter. Univariate analysis with probe set 20862_s_at (*YBX1*) using auto-selected cutoff and excluded biased arrays. **(E)** High *YBX1* expression correlates with poor prognosis in MPM patients (*p* = 8.6 × 10^–3^). Kaplan-Meir plot was generated using PROGgene V2 with the TCGA mesothelioma dataset (*n* = 83) using “DEATH” as the survival measure and median as the cutoff.

As for MPM, next-generation sequencing of 216 MPM patients showed that the tumor suppressors *BAP1*, *NF2*, and *SETD2* were significantly mutated through gene fusions and splicing alterations (Bueno et al., 2015). *CDKN2A*, which encodes the tumor suppressor p16^INK4a^, is also frequently deleted in up to 75–90% of MPM cases (Ladanyi, 2005; Sementino et al., 2018). Data from TCGA reflects the above findings, apart from *ALK* alterations in ADC, which were present in 7% of cases (Figures 1A–C). An important distinction must between lung cancer and MPM is that lung cancers are generally characterized by an increase in oncogenic drivers, while MPM appears to be more commonly defined by loss of tumor suppressors (Ladanyi, 2005; Bueno et al., 2015; Jamal-Hanjani et al., 2017; Figure 1C). This makes identifying new therapeutic targets in MPM more challenging. Apart from bevacizumab, which targets vascular endothelial growth factor A, no targeted therapies are currently available to MPM patients (Brosseau et al., 2017).

The story for SCLC patients is similar with no breakthrough changes in treatment in over 25 years despite decades of research. The only exception to this is the approval of topotecan as a second-line therapy (Hirsch et al., 2017), and immunotherapy, which has shown some promise in Phase I/II trials in PD-L1 positive relapsed SCLC patients (Ott et al., 2015). Unfortunately, immunotherapy success has been limited by rapid disease progression, which can result in patient death before an effective anti-tumor response has time to occur (3–6 months), and severe immuno-related toxicities (encephalitis or myasthenia gravis) that are already highly associated with SCLC (Oronsky et al., 2017). Other drugs such as PARP inhibitors and transcription inhibitors have shown some preclinical promise, but have yet to translate into clinical benefits for SCLC patients (Oronsky et al., 2017).

For NSCLC, targeted therapies have provided promising, albeit limited, results. The best known of these are the EGFR tyrosine kinase inhibitors such as erlotinib and osimertinib, which have proved effective for EGFR mutant ADC tumors (Hirsch et al., 2017; Winther-Larsen et al., 2019). In the ADC TCGA dataset, 21% of patients had EGFR alterations (Figure 1A), although the occurrence of EGFR mutations can vary between populations in ADC and NSCLC as a whole. For example, while EGFR mutation can occur in up to 40% of all NSCLC patients of Asian descent, the frequency of mutation in non-Asian NSCLC populations drops to 10–20% (Hirsch et al., 2017). Another problem is that response to EGFR inhibitors is almost always followed by the emergence of resistance (Hirsch et al., 2017). ALK inhibitors are similarly effective in patients harboring ALK translocations (Hirsch et al., 2017), present in 2% of all NSCLC patients (Hirsch et al., 2017). Alterations of ALK in ADC tumors specifically is found in up to 7% of cases, according to TCGA data (Figure 1A). Inhibitors targeting BRAF mutant tumors (3–5% of lung cancers), MET overexpressing tumors (3–4% of ADC cases) and tumors harboring RET fusion proteins (1–2% of NSCLC) are also currently undergoing preclinical and clinical studies (Hirsch et al., 2017). The remaining majority of patients with ADC have no known actionable targets.

Patients with SCC have even fewer options with only ∼13% of SCC tumors reported to harbor at least one currently actionable alteration (Lindquist et al., 2017). There is no subset of patients known to benefit from targeted drugs at the moment, although there is some benefit from immunotherapy (Hirsch et al., 2017; Friedlaender et al., 2019). *TP53* mutations are a common alteration in SCC patients (33%; Figure 1B), however, existing targeted *TP53* treatments have proven ineffective (Friedlaender et al., 2019). *PIK3CA* is also frequently altered in thoracic cancer, particularly in SCC (Friedlaender et al., 2019) (71%; Figure 1B), indicating that it may have significance as a therapeutic target. However, despite promising preclinical studies of PIK3CA inhibitors, the benefit of these drugs appears to be negligible in trials with NSCLC patients (Friedlaender et al., 2019). This has also been the case in other cancers where, generally, patients show limited response and many experience prohibitive toxicity (Janku et al., 2018). This pathway mediates a multitude of downstream effects, which may attest to the observed relative ineffectiveness of PIK3CA inhibitors in lung cancer. *FGFR1* amplification occurs in 20–25% of SCC cases (Friedlaender et al., 2019; Figure 1B), but again, targeting it in the clinic has provided limited efficacy and its potential as a viable target remains under contention (Hirsch et al., 2017; Friedlaender et al., 2019). There are a few targets that have been the focus of preclinical studies showing promising results, such as the transcription factor SOX2. SOX2 is involved in cell lineage-survival (Friedlaender et al., 2019) and is often upregulated in SCC (Karachaliou et al., 2013; Friedlaender et al., 2019) (60%; Figure 1B), as well as SCLC (Rudin et al., 2012; Karachaliou et al., 2013) and to a lesser degree ADC (Karachaliou et al., 2013). Finding such targets and translating them to the clinic is essential to improve outcomes for patients with SCC.

The heterogeneity of thoracic cancer biology makes finding clinically relevant therapeutic targets inherently difficult. Identifying other penetrant driver events in thoracic cancers may uncover alternative targets, which could yield more therapeutic options for patients down the line. One such potential target is YB-1. YB-1 is downstream of the commonly dysregulated PI3K-AKT-mTOR pathway, so targeting it may refine the effects of inhibiting this signal cascade. Thus, anti-YB-1 agents may provide more tumor-specific results than their upstream-targeting counterparts, such as PIK3CA inhibitors (Janku et al., 2018). Adding to this, YB-1 upregulates PIK3CA at the transcriptional level in breast cancer (Astanehe et al., 2009). This implies that YB-1 may be involved in a feed-forward loop with the PI3K-AKT pathway and that targeting it could be an effective strategy in tumors with heightened PIK3CA, such as SCC. YB-1 is also upstream of SOX2 (Jung et al., 2014) and a host of other oncogenic drivers (Lasham et al., 2013), so the downstream effects of YB-1 inhibition may still be broad enough to make it an interesting candidate. This review therefore outlines recent literature focusing on YB-1 in cancer and makes the case for its possible use as a biomarker and future therapeutic target in thoracic malignancies.

### Y-Box Binding Protein-1 in Thoracic Cancers: An Overlooked Target?

Y-box binding protein-1, encoded by the *YBX1* gene, is a multifunctional oncoprotein involved in many hallmarks of cancer development including driving proliferation, invasion and metastasis, CSC biology, resistance to chemotherapy, hypoxic response, DNA repair and exosomal sorting. Despite these links YB-1 has received limited attention as a therapeutic target or biomarker in oncology (Lasham et al., 2013). Although mutations of *YBX1* are rare [∼1% in all cancers types (Cerami et al., 2012; Gao et al., 2013)], overexpression of YB-1 is found in a wide range of cancers and is often associated with poor prognosis (Lasham et al., 2013), including NSCLC and MPM. Analysis of TCGA data shows that elevated *YBX1* expression was highly prognostic in a cohort of 1,926 NSCLC patients (Győrffy et al., 2013; Figure 1D) and in 83 mesothelioma patients (Goswami and Nakshatri, 2014; Figure 1E). This supports the results of a recent meta-analysis of data from 692 NSCLC patients which found that high YB-1 protein expression significantly correlated with poorer overall survival and clinicopathological features (Jiang et al., 2017). YB-1 is overexpressed in mesothelioma compared to non-malignant mesothelial cells *in vitro* (Johnson et al., 2018) and a small study of 33 MPM patients showed a trend toward higher YB-1 expression in sarcomatoid MPM tumors, which are associated with shorter survival (Iwanami et al., 2014). Unfortunately, TCGA data is currently not available for SCLC, likely because surgically resected tissue specimens are relatively rare (Byers and Rudin, 2015) and, to our knowledge, a prognostic study on YB-1 expression in SCLC is yet to be conducted.

In the above datasets, alterations were seen in 12, 11, and 8% in ADC, SCC, and MPM, respectively, and mRNA upregulation was predominant (Figures 1A–C). While only PIK3CA and SOX2 were significantly co-expressed in the SCC dataset (*q* < 0.001; Figure 1B), notably, this analysis did not show *YBX1* alteration to be significantly associated with the current targetable oncogenes *ALK*, *BRAF* or *EGFR* in ADC (Figure 1A), despite there being a small proportion of tumors that had elevated levels of both *YBX1* and *EGFR*. This suggests that YB-1 deregulation may represent a unique subpopulation of patients that may not have a targetable mutation. This combined with the prognostic significance of YB-1 in NSCLC and MPM, suggests that YB-1 may be a clinically relevant target worthy of further investigation.

## YB-1: A Malignant Jack of All Trades

### A Driver of Malignant Phenotypes

Y-box binding protein-1 was first discovered as a negative transcriptional factor of major histocompatibility complex Class II where it binds to the Y-box (5′-CTGATTGG-3′) (Didier et al., 1988). Further investigation found that YB-1 stimulated the transcription of a wide variety of genes, including important oncogenes such as *EGFR* and HER2 (Lasham et al., 2013). YB-1 is a part of the cold-shock protein superfamily and contains a conserved nucleic acid binding region termed the CSD (Wolffe et al., 1992; Figure 3). Along with the CSD, YB-1 is comprised of two other highly disordered domains, the alanine/proline rich variable N–terminal domain and the C–terminal domain (CTD), each facilitating different biological interactions (Lyabin et al., 2014; Suresh et al., 2018). This versatility affords YB-1 a range of functions including transcriptional regulation, DNA repair and pre-mRNA splicing (Lyabin et al., 2014). YB-1 is also a major component of messenger ribonucleoprotein complexes and is integrally involved in mRNA stabilization and the translational activation or repression of many genes (Suresh et al., 2018). This assortment of functions manifest themselves in an equally broad spectrum of biological roles in cancer (Lasham et al., 2013; Lyabin et al., 2014). The general cancer-related activities of YB-1 have been previously reviewed (Lasham et al., 2013; Kosnopfel et al., 2014; Lyabin et al., 2014) and therefore we will primarily focus on recent publications on the role of YB-1 specifically in lung cancer and MPM here. The evidence supporting each phenotype driven by YB-1 and the relevant interaction partners for the following sections is summarized in Table 1.

**TABLE 1:** Roles and interaction partners of YB-1 related to thoracic cancer biology.

**Phenotype**	**Role in thoracic cancer behavior**	**Targets or interactions**	**Other cancers/evidence**
Proliferation and cell cycle progression	Knockdown induces growth inhibition of NSCLC (Harada et al., 2014; Zhao et al., 2016) and MPM (Johnson et al., 2018)	Transcriptional regulation of *EGFR* (Hyogotani et al., 2012), E2F family members (Lasham et al., 2011), *CCND1* (Harada et al., 2014) and *CDC25A* (Zhao et al., 2016)	Basal-like breast cancer (Stratford et al., 2007) spinal chordoma (Liang et al., 2019)
Migration, EMT, invasion and metastasis	Overexpression in lung ADC promotes E- to N-cadherin shift, EMT and migration (Ha et al., 2015) Knockdown inhibits invasion and metastasis of lung cancer cells (Zhao et al., 2016; Guo et al., 2017) Knockdown inhibits migration and invasion of MPM cells (Johnson et al., 2018)	Translational activation of *SNAI1* (Evdokimova et al., 2009a, b) Involvement in E/M related Wnt signaling – β-catenin (Chao et al., 2017)	Breast cancer (Lim et al., 2017), melanoma (Jia et al., 2017), nasopharyngeal cancer (Zhou et al., 2017b), skin squamous cell carcinoma (Wang W. et al., 2017) spinal chordoma (Liang et al., 2019) Overexpression induces E/M phenotype (Gopal et al., 2015)
Cancer stem-like cells	Drives metastatic CSC-like properties in lung cancer (Guo et al., 2017)	Transcriptional regulation of *SOX2* (Jung et al., 2014; Bledzka et al., 2017), *NANOG* (Bledzka et al., 2017; Chao et al., 2017; Guo et al., 2017) and *Oct4* (Bledzka et al., 2017; Chao et al., 2017)	Hepatocellular carcinoma (Chao et al., 2017), brain (Mantwill et al., 2013), osteosarcoma (Xu et al., 2015), and breast (Davies et al., 2015) CSCs
Hypoxic response	*Requires further investigation*	Translational regulation of *HIF1*α(El-Naggar et al., 2015) and *FOXO3a* (Emerling et al., 2008; Chou et al., 2015) Transcriptional repression of *EPO* (Rauen et al., 2016)	Translocation to nucleus under hypoxic stress (Rauen et al., 2016)
LRP downregulation after YB-1 knockdown and correlation with LRP (Hyogotani et al., 2012) response	LRP downregulation after YB-1 knockdown and correlation with LRP (Hyogotani et al., 2012)	Transcriptional regulation of *LRP* (Stein et al., 2005) and *MRP1* (Stein et al., 2001; Mantwill et al., 2006)	Neuroblastoma (Wang H. et al., 2017), esophageal SCC (Xu and Hu, 2016), bladder cancer (Shiota et al., 2011), melanoma (Schittek et al., 2007), ovarian cancer (Yahata et al., 2002)
DNA repair	Involved in cigarette-smoke induced guanine oxidization prevention and correlations in COPD patients (Deslee et al., 2010)	Complex with PCNA at cisplatin-modified DNA (Ise et al., 1999; Gaudreault et al., 2004) PARP1 poly(ADP-ribosyl)ation of YB-1 (Alemasova et al., 2015) Scaffold for BER proteins (Dutta et al., 2015; Alemasova et al., 2016) Scaffolds for XPC (NER protein) (Fomina et al., 2015)	Preferential binding to cisplatin-modified DNA (Ise et al., 1999)
Exosomes	*Requires further investigation*	ncRNA (Shurtleff et al., 2017; Suresh et al., 2018)	Presence in non-malignant and malignant exosomes (Shurtleff et al., 2017; Suresh et al., 2018) Role in exosomal ncRNA sorting (Shurtleff et al., 2017; Suresh et al., 2018)

### A Promoter of Cell Proliferation and Cell Cycle Progression

The proliferative role of YB-1 in cancer has been demonstrated in many malignancies, driven by its regulation of highly penetrant downstream oncogenic growth promoting genes (Lasham et al., 2013). A prime example is the transcriptional activation of *EGFR* by YB-1. A study of 105 NSCLC samples showed that YB-1 and EGFR were significantly co-expressed and knockdown of YB-1 in two NSCLC cell lines resulted in reduction of EGFR (Hyogotani et al., 2012). Similar results have also been observed in basal-like breast cancer and spinal chordoma (Stratford et al., 2007; Liang et al., 2019). Notably, overexpression of EGFR in lung cancer and mesothelioma promotes cell growth, invasion and angiogenesis (Ciardiello et al., 2004; Destro et al., 2006). Several cell cycle regulators are also under YB-1 control, including the E2F family. YB-1 specifically binds to the promoter of cell cycle activators transcription factor *E2F1* and transcription factor *E2F2* and YB-1 knockdown reduced cell proliferation of a NSCLC cell line *in vitro* and *in vivo* (Lasham et al., 2011). In NSCLC cells, YB-1 transcriptionally activates *CCND1* a protein critical for progression through the G1 phase (Harada et al., 2014). YB-1 also binds to and activates the promoter of the dual specific phosphatase *CDC25A*, driving G1/S cell cycle progression (Zhao et al., 2016). These studies demonstrate the important role of YB-1 by showing that its knockdown with siRNA induces G0/G1 cell cycle arrest *in vitro* and *in vivo* (Harada et al., 2014; Zhao et al., 2016). Similarly, we have also shown that targeting YB-1 with siRNA can inhibit the growth of MPM cells *in vitro* (Johnson et al., 2018).

Y-box binding protein-1-driven proliferation may require a region within its N–terminal domain. Breast cancer cells overexpressing a YB-1 CTD fragment (from amino acid 125 onward) exhibited proliferation inhibition *in vitro* and *in vivo* (Shi et al., 2016). It is possible that the removal of Ser102, a site commonly phosphorylated and associated with growth (discussed further in section “Post-Translational Modification in the Control of YB-1 Activity and Localization”), could explain the lack of growth promotion here. However, as growth was actively inhibited in response to the upregulation of the YB-1 CTD, this could also suggest that YB-1, or certain regions of it, may inhibit proliferation under specific gene dosages or biological contexts. For example, YB-1 overexpression in Ras-MAPK activated breast cancer cells led to YB-1-mediated translational repression of growth-promoting genes, lowering proliferation rates. This was accompanied by the induction of EMT-like changes which promoted migration, invasion and allowed cells to survive in anchorage-independent conditions (Evdokimova et al., 2009b). This suggests that YB-1 expression levels determine its function, driving either a proliferative or invasive phenotype.

### YB-1 Is a Central Player in EMT, Invasion and Metastasis

Invasion and metastasis are key behaviors of lung cancer and mesothelioma cells that contribute to patient death and the poor prognosis observed with these tumors. YB-1 is known to play a role in the migration of thoracic cancer cells. Stable overexpression of YB-1 in lung ADC cells induced E-cadherin downregulation, N-cadherin upregulation, accelerated TGFβ1-induced EMT and cell migration (Ha et al., 2015). In support, silencing YB-1 inhibited the invasion and metastasis of lung cancer cells *in vitro* and *in vivo* (Guo et al., 2017). YB-1 overexpression also significantly increased the invasive capacity of these cells *in vitro* (Guo et al., 2017). Similarly, knockdown of YB-1 inhibited lung cancer migration (Zhao et al., 2016) and MPM migration and invasion (Johnson et al., 2018) *in vitro*. YB-1 has also been implicated in the migration and invasion of breast cancer (Lim et al., 2017), melanoma (Jia et al., 2017), nasopharyngeal cancer (Zhou et al., 2017b), skin squamous cell carcinoma (Wang W. et al., 2004) and spinal chordoma (Liang et al., 2019).

Epithelial-mesenchymal transition is thought to be a primary mechanism facilitating cancer cell invasion and metastasis through inducing phenotypic plasticity (Brabletz, 2012). Current evidence suggests that EMT is a progressive, transient and reversible process and that cells in a hybrid E/M state - partial EMT – exhibit significantly higher tumorigenic potential compared to exclusively epithelial or mesenchymal cells (Pastushenko et al., 2018; Kröger et al., 2019).

Hybrid epithelial/mesenchymal state hybrids can be promoted by Zinc finger protein SNAI1 (Snail, gene *SNAI1*) transcription factor activity, the expression of which is specific to E/M populations of basal breast cancer cells (Kröger et al., 2019). Snail protein was found to be 5-fold higher in such cells compared to mesenchymal populations, while epithelial cells displayed undetectable levels (Kröger et al., 2019). However, this was only accompanied by a 1.5-fold increase in *SNAI1* transcript expression, implying that translational activation is more important in Snail overexpression than transcriptional regulation (Kröger et al., 2019). YB-1 translationally upregulates Snail expression (Evdokimova et al., 2009a, b), suggesting that YB-1 could also be a key promoter the E/M state. In support, stable YB-1 overexpressing epithelial Madin-Darby canine kidney (MDCK^YB–1^) cells exhibited a partial EMT-like phenotype and establish viable tumor xenografts in mice, while parental MDCK cells did not (Gopal et al., 2015). This increased tumorigenicity was also accompanied by elevated secretion of angiogenic factors (Gopal et al., 2015). Treatment of endothelial cells with concentrated conditioned medium from MDCK^YB–1^ cells also stimulated cell migration (Gopal et al., 2015).

Wnt signaling is also a primary driver of partial and complete EMT. β-catenin-dependent canonical Wnt signaling is thought to be preferentially active in E/M populations (Reya and Clevers, 2005; Kröger et al., 2019), while β-catenin-independent non-canonical signaling is more associated with a mesenchymal state, migration and invasion (Weeraratna et al., 2002; Gujral et al., 2014). Knockdown of YB-1 in hepatocellular carcinoma cells disrupted stemness and suppressed β-catenin protein expression and nuclear translocation, which was rescued by overexpression of the active form of β-catenin (Chao et al., 2017). This regulation of β-catenin-dependent Wnt signaling further supports a potential role for YB-1 in driving a partial EMT state. Interestingly, populations in the partial EMT state are also enriched with CSCs (Kröger et al., 2019), suggesting that YB-1 may also play are role in regulating these important cancer progenitors.

### Involvement in Cancer Stem-Like Cells

Cancer stem-like cells are becoming recognized as important drivers of disease progression and are thought to be a major contributing factor toward metastasis, the development of drug resistance and recurrence of most cancers, including those of the thorax (Leon et al., 2016; MacDonagh et al., 2016; Makena et al., 2018). CSCs are a heterogeneous, slow growing population of cells within a tumor. They have self-renewal ability but one subpopulation, termed metastatic CSCs, can disseminate through blood vessels and initiate metastasis (Dalerba and Clarke, 2007). This was clearly demonstrated in pancreatic cancer, where eradicating the metastatic CSC population dramatically reduced metastatic but not tumorigenic potential, implying that a subgroup of CSCs are responsible for metastasis (Hermann et al., 2007).

One recent study has shown that YB-1 enforces lung cancer metastatic CSC-like properties *in vitro* and *in vivo* through transcriptional upregulation of *NANOG*, a marker of CSCs required for the invasion and sphere formation of ADC cells *in vitro* (Guo et al., 2017). Supporting this, knockdown of YB-1 in hepatocellular carcinoma cells reduced *NANOG* and *Oct4*, as well as α-fetoprotein transcript expression (Chao et al., 2017). This follows findings showing NANOG and Oct4 are upregulated in ADC, which induce sphere formation, drug resistance and EMT (Chiou et al., 2010). YB-1 also regulates SOX2 in breast CSCs, maintaining stem-like properties and tumorigenic potential (Jung et al., 2014). Given the probable importance and frequent upregulation of SOX2 in lung cancer (Rudin et al., 2012; Karachaliou et al., 2013; Friedlaender et al., 2019; Figure 1B), a study investigating the relationship between YB-1 and SOX2 in thoracic cancer may further implicate YB-1 in the biology of these diseases.

Y-box binding protein-1 has been shown to be important in other cancer CSCs as well. Brain CSCs were shown to have high expression of YB-1 which was utilized in a YB-1-based virotherapy *in vitro* (Mantwill et al., 2013). The re-expression of the microRNA miR-382 in osteosarcoma cells significantly decreased the CSC population resulting in reduced relapse after doxorubicin treatment, EMT and metastasis both *in vitro* and *in vivo* (Xu et al., 2015). The authors attributed these tumor suppressive functions of miR-382 to targeting and downregulating YB-1 (Xu et al., 2015). This microRNA is downregulated in NSCLC and exogenous miR-382 expression inhibits NSCLC growth, migration and invasion via the suppression of *SETD2* (Chen T. et al., 2017) and *LMO3* (Chen et al., 2019). In breast cancer, inhibition of p90 RSK, a major kinase involved in YB-1 phosphorylation; see section “Post-Translational Modification in the Control of YB-1 Activity and Localization”) using the small molecule LJI308 eradicated the population of breast CSCs and induced apoptosis in breast cancer cells (Davies et al., 2015). RSK is thought to have potential as a therapeutic target as it is involved in the proliferation of lung cancer (Poomakkoth et al., 2016). Furthermore, knockdown of WAVE3, a protein required for nuclear translocation of YB-1, prevented YB-1 mediated transcriptional activation *NANOG*, *SOX2* and *Oct4* in breast CSCs (Bledzka et al., 2017). WAVE3 expression was also correlated with that of YB-1 and more aggressive phenotypes of breast cancer (Bledzka et al., 2017).

### YB-1 Is Involved in Hypoxic Response

The maintenance of CSCs is intertwined with the effects of hypoxia (Li and Rich, 2010). Supporting its role in thoracic CSC biology, hypoxia promotes an aggressive phenotype in MPM and upregulates Oct4, a marker of CSCs (Kim et al., 2018). Oct4 is also important in gefitinib-resistant lung CSCs and cisplatin-induced stemness in NSCLC has been linked to hypoxia-inducible factors (Kobayashi et al., 2016). Hypoxia occurs in most solid tumors and has been linked to CSC maintenance and behavior (Li and Rich, 2010; Bao et al., 2012), as well as disorganized tumor vascularization, EMT and metastasis (Muz et al., 2015). Factors such as HIF1α drive hypoxia-mediated transcription, influencing cell immortalization, metastasis and vascularization (Semenza, 2014). YB-1 translationally regulates HIF1α (El-Naggar et al., 2015) and acts as a transcriptional repressor for the HIF1α inhibitor *FOXO3a* via competition for p300 during vascular development (Emerling et al., 2008; Chou et al., 2015). Under hypoxic conditions YB-1 translocates to the nucleus where it binds to hypoxia response elements within the 3′ enhancer of the *EPO* gene and blocks its expression (Rauen et al., 2016). Hypoxia plays an important role in driving malignant cellular behavior, including resistance to chemotherapy (Rohwer and Cramer, 2011). While YB-1-driven response to hypoxia may contribute toward chemoresistance, its activity as a transcription factor may also play a role in drug inefficacy.

### A Possible Role for YB-1 in Resistance to Platinum-Based Chemotherapy

Although the role of YB-1 has not yet been studied in lung cancer or MPM, it has been shown to be involved in the chemoresistance of many cancers including that of platinum-based chemotherapies (To et al., 2010; Kang et al., 2013; Lasham et al., 2013; Shiota et al., 2014; Yamashita et al., 2017). Silencing YB-1 induces cisplatin sensitization in neuroblastoma (Wang H. et al., 2017), esophageal SCC (Xu and Hu, 2016), bladder cancer (Shiota et al., 2011) and melanoma (Schittek et al., 2007). Treatment with cisplatin also stimulates YB-1 production in bladder cancer (Shiota et al., 2010), while ovarian cancer cells with acquired cisplatin resistance show an increase in nuclear YB-1 expression (Yahata et al., 2002), suggesting that cancer cells may increase YB-1 production as a protective measure. The reasons why YB-1 may provide protection are still unclear. However, YB-1 does transcriptionally upregulate *LRP*, aka *MVP* (Stein et al., 2005), the principal component of vaults in human cells. Vaults are highly conserved ribonucleoproteins which have been suggested to play a role in the resistance of cancer cells to cisplatin, among other chemotherapies, by sequestering drugs away from their intended targets (Wang W. et al., 2004; Lara et al., 2011). YB-1 knockdown in lung cancer cell lines resulted in LRP downregulation and nuclear staining of YB-1 correlated with LRP expression in 105 NSCLC samples, conferring significantly lower overall survival (Hyogotani et al., 2012). However, this study did not investigate the effect of this knockdown on the chemoresistance of any drug.

Y-box binding protein-1 has also been linked to *MRP1* gene activation (Stein et al., 2001; Mantwill et al., 2006), an efflux ATP-binding cassette transporter which is thought to contribute toward multidrug resistance (Stefan and Wiese, 2019). High levels of LRP and MRP1 correlated with lower response to cisplatin chemotherapy, poorer progression free survival and overall survival in advanced NSCLC patients receiving cisplatin-based chemotherapy (Li J. et al., 2009; Li X.Q. et al., 2009). Treatment with cisplatin also induces heightened LRP expression in ADC and SCC cell lines (Xu et al., 2017a) and *LRP* gene expression was significantly increased compared to control pleura samples in a study of MPM patients (Singhal et al., 2003). *MDR1* gene (encoding P-glycoprotein 1), which is dependent on the nuclease and base excision repair enzyme APE1 expression, has also been implicated in YB-1-driven cisplatin resistance (Ohga et al., 1998; Chattopadhyay et al., 2008). However, the evidence supporting a clear role for P-glycoprotein 1 as an integral player in the chemoresistance of lung cancer and mesothelioma remains contentious, implying that other targets may be more important (Soini et al., 2001; Wangari-Talbot and Hopper-Borge, 2013).

### An Agent of DNA Repair in Response to Cisplatin and Oxidative Stress

Y-box binding protein-1 may drive chemoresistance through the upregulation of the above targets and through driving a hypoxic response. However, some of its other functions may also contribute, such as its role as part of the DNA repair machinery. Oxidative stress and resulting chronic inflammation has long been implicated as a primary driver of cigarette smoking-related diseases, including lung cancer (Park et al., 2009; Sears, 2019). Altered DNA repair pathways have been implicated in the carcinogenesis of lung cancer in response to cigarette smoke-related DNA damage, particularly the NER and BER pathways (Sears, 2019). There is also a body of evidence supporting the suggestion that COPD leads to the development of lung cancer, or at least that the two are correlated (Sears, 2019). Chronic inflammation caused by asbestos-related oxidative stress is a major driver of MPM carcinogenesis (Benedetti et al., 2015; Chew and Toyokuni, 2015), implying that aberrations in DNA repair machinery in response to oxidization play a role in the progression of many thoracic cancers.

Y-box binding protein-1 has been suggested to be part of the DNA repair machinery as it binds to enzymes involved in BER, mismatch repair and DNA double-stranded break repair, previously reviewed (Alemasova and Lavrik, 2017). YB-1 binds preferentially to cisplatin-damaged DNA complexed with PCNA, where it works to separate cisplatin-damaged DNA strands, recruit DNA repair proteins and displays weak endonucleolytic and exonucleolytic function (Ise et al., 1999; Gaudreault et al., 2004). PARP1 has also been shown to catalyze the poly(ADP-ribosyl)ation of YB-1 in the presence of DNA damage, further supporting a role for YB-1 in DNA repair (Alemasova et al., 2015).

Y-box binding protein-1 is also involved in NER and BER in response to oxidative stress. DNA damage-related stress stimulates YB-1 nuclear translocation (Cohen et al., 2010) (discussed further in section “Control of YB-1 Subcellular Localization”) where it can bind to oxidized DNA lesions, structurally altering DNA to allow access to the damaged site while recruiting and scaffolding proteins involved in BER including PARP1, PARP2, NEIL1, and PCNA, among others (Dutta et al., 2015; Alemasova et al., 2016). In ssDNA, YB-1 suppresses NEIL1-mediated apurinic/apyrimidinic site cleavage, and it has been suggested that the role of YB-1 in DNA repair can prevent ssDNA breaks and induce oxidative nucleotide repair in double-stranded DNA (Dutta et al., 2015). YB-1 has also been linked to NER. Cross-talk between YB-1 and XPC (an important player in NER which has significance in lung cancer carcinogenesis and is affected by germline mutation in MPM), results in their assembly at DNA damage sites (Jin et al., 2014; Fomina et al., 2015; Betti et al., 2017; Sears, 2019).

Y-box binding protein-1 was found to be involved in mitigating cigarette smoke-induced guanine oxidization in lung fibroblasts and mice chronically exposed to cigarette smoke, and that lung samples of late-stage COPD patients exhibited significantly lower YB-1 levels compared to early mid stage patients or patients without COPD (Deslee et al., 2010). The role YB-1 plays in DNA repair (particularly from oxidization) and the fact that it is secreted under oxidizing conditions (see section “YB-1 is Secreted Into the Extracellular Space Under Cellular Stress”) implies that YB-1 may promote the oxidation-related carcinogenesis of lung cancer and MPM. Cigarette-induced oxidative stress has additionally been suggested to induce the release of exosomes (Ryu et al., 2018), the sorting of which are also mediated in part by YB-1.

### YB-1 and Exosomal RNA Sorting

Extracellular vesicles such as exosomes are used by cells for intercellular communication to both their immediate and distant surroundings (Mashouri et al., 2019). Exosomes carry factors such as proteins, mRNA and miRNA to mediate processes including embryonic development, injury response and homeostasis (Mashouri et al., 2019). Exosomes also play versatile and key roles in cancer cell behavior and remodeling of the tumor microenvironment (Mashouri et al., 2019). A malignant role for exosomes in lung cancer is well documented, where exosomes can induce proliferation, angiogenesis, EMT changes and metastasis (Vanni et al., 2017; Zhou et al., 2017a; Ryu et al., 2018). Exposure to cigarette smoke is also thought to induce the release of extracellular vesicles, such as exosomes, which has been linked to the development of COPD and possibly the development of lung cancers (Ryu et al., 2018). Asbestos exposure also alters the exosomal cargo of lung epithelial cells *in vitro* and exposing non-malignant mesothelial cells to these exosomes induces gene expression changes related to EMT and other cancer related pathways (Munson et al., 2018). This indicates that exosomes may play an integral role in the carcinogenesis of mesothelioma. MPM cell lines also secrete higher levels of exosome-associated proteins linked to stress response and proliferation compared to their non-malignant counterparts (Creaney et al., 2017). Supporting this, exosomes from MPM cells have a distinct oncogenic signature and stimulate the migration of fibroblasts and endothelial cells (Greening et al., 2016).

Y-box binding protein-1 is known to be involved in exosomal RNA-sorting, reviewed previously (Suresh et al., 2018), which may indicate it is involved in altering malignant exosomal expression profiles. Briefly, the presence of YB-1 in exosomes has been shown in both malignant and non-malignant cells alike where it helps to define the levels of several RNA species, including miRNA and tRNA (Shurtleff et al., 2017; Suresh et al., 2018). However, to our knowledge no study has investigated YB-1 in lung cancer and mesothelioma exosomal sorting. Future studies following this line may shed further light into the underlying mechanisms of exosomes and their role in thoracic cancer biology.

### A Role in Immune Evasion?

Evidence in other tumor types suggests that the upregulation of YB-1 could drive immune evasion. For example, in doxorubicin-resistant hepatocellular carcinoma cells, YB-1 is overexpressed, which in turn transcriptionally upregulates the expression of PD-L1 and decreases the secretion of the chemokines IL1β, IL10, and TGFβ *in vitro* (Tao et al., 2019). High YB-1 was also associated with resistance to cisplatin, gemcitabine, docetaxel, dasatinib and gefitinib in this study (Tao et al., 2019). This suggests that resistance to these drugs may also result in heightened PD-L1 and subsequent immunosuppression via YB-1 upregulation, at least in hepatocellular carcinoma. In light of these results, investigating the potential of a similar role in thoracic cancers would be of great interest.

### YB-1 Regulation: A Complex Network of Transcriptional, Translational and Post-translational Control

The wide-ranging roles of YB-1 in cell biology imply that its expression, localization and function must be tightly regulated in normal physiology. As YB-1 is frequently overexpressed in cancer, dysregulation of these controlling systems may play a role in malignant transformation. The expression and localization of YB-1 is controlled by a complex network of transcriptional, translational and autoregulatory signals discussed below.

### Transcriptional Control

**FIGURE 2:**
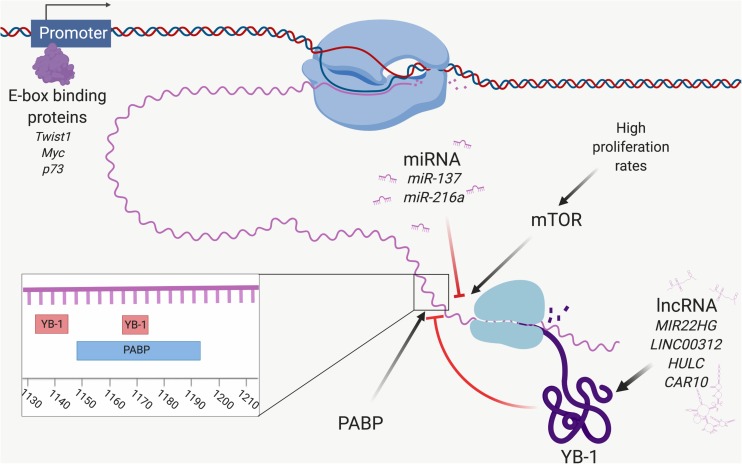
Control of YB-1 expression. A network of factors controls *YBX1* expression at the transcriptional and translational levels. The E-box binding proteins Twist1, Myc and p73 interact with the promoter of *YBX1* and initiate transcription of *YBX1* mRNA. *YBX1* mRNA expression is downregulated by targeting miRNA, including miR-137 and miR-216a. *YBX1* translation is stimulated by mTOR, which itself is influenced by proliferation rate. YB-1 protein function and expression are modulated by lncRNA, including MIR22HG and LINC00312. YB-1 is involved in an autoregulatory feedback loop and binds to *YBX1* mRNA at two sites (nucleotides 1133–1145 and 1165–1172), inhibiting its own translation. PABP stimulates *YBX1* translation by binding to a site located at 1149–1196, overlapping the second YB-1 binding site. Poly(A)-binding protein (PABP) and YB-1 compete for this site and hence regulate the level of YB-1 protein expression. Created with Biorender.com.

Several transcription factors have been found to promote YB-1 transcription by binding to motifs in the *YBX1* promoter. For example, *YBX1* transcription has been shown to be promoted by GATA transcription factors, although recent evidence suggests the GATA family is less important for promoting *YBX1* expression in ADC (Yokoyama et al., 2003; Murugesan et al., 2018). Possibly more important are the six E-boxes located in the promoter of *YBX1* (Makino et al., 1996). The first is located at 48–53 nucleotide residues away from the promoter, the second at 353–358, the third at 458–463, the fourth at 531–536, the fifth at 1147–1152, and the sixth at 1201–1206 (Makino et al., 1996). The E-box binding transcription factor Twist1 also stimulates *YBX1* transcription, driving cell growth and EMT (Shiota et al., 2008; He et al., 2015; Figure 2). A recent meta-analysis of 572 NSCLC patients showed that high Twist1 expression significantly correlated with poorer patient prognosis, recurrence-free survival and lymph node or other metastasis (Li et al., 2018). A small retrospective study of mesothelioma samples also showed that Twist1 expression was significantly higher in sarcomatoid tumors (expressed in 7/7 of samples) compared to biphasic (6/10) and epithelioid tumors (7/17) (Iwanami et al., 2014). Although the percentage of samples positive for YB-1 was almost identical to that of Twist1 in this study (6/7 in sarcomatoid, 6/10 in biphasic and 7/17 in epithelioid), whether YB-1 and Twist1 were co-expressed in the same samples was not determined (Iwanami et al., 2014).

An E-box within the YB-1 promoter is also trans-activated by Myc and p73 to drive the transcription of *YBX1* (Uramoto et al., 2002; Figure 2). The ability of Myc to transcriptionally activate *YBX1* is interesting, not only as Myc drives malignant behavior and is often associated with poor prognosis in thoracic cancers (Jiang et al., 1992; Volm and Koomagi, 2000; Riquelme et al., 2014), but because YB-1 can itself initiate *Myc* translation by acting as a specific internal ribosome entry segment-trans-activating factor (Cobbold et al., 2010). YB-1 was also shown to regulate *Myc* at the transcriptional level in bladder cancer, with implications on aerobic glycolysis (Warburg effect) (Xu et al., 2017b). This feed forward loop was first described in multiple myeloma (Bommert et al., 2013), however, it is quite possible that a similar feed forward loop accounts for both YB-1 and Myc overexpression in thoracic cancers, driving malignant progression and aggressiveness.

### Translational Regulation of YB-1

Y-box binding protein-1 expression is also regulated at the translational level, most notably via signaling through the mTOR pathway (Figure 2), which regulates cell growth, motility, survival, transcription and protein synthesis via the integration of signals from hormone and growth factor stimulation, availability of nutrients, and stress (Zarogoulidis et al., 2014). mTOR signaling promotes the translation of *YBX1* and increases the phosphorylation of RSK, a serine/threonine kinase which phosphorylates and thereby activates YB-1 (Mendoza et al., 2011; Lyabin et al., 2012). RSK has been implicated in lung cancer proliferation and has itself been suggested as a target with therapeutic significance (Poomakkoth et al., 2016).

The division rate of eukaryotic cells affects *YBX1* translation via mTOR regulation. Slow dividing and serum-starved cell populations exhibit attenuated mTOR signaling, which in turn inhibits *YBX1* translation (Lyabin et al., 2012). This pathway is frequently activated in lung cancer and antagonizing mTOR in such cells has proven to be a potential therapeutic avenue (Zarogoulidis et al., 2014). The PI3K/mTOR pathway is also highly activated in mesothelioma, but not in non-malignant mesothelial cells (Zhou et al., 2014) or adjacent tissue (Hoda et al., 2011), and phospho-mTOR was significantly associated with poorer overall survival in a cohort of 107 mesothelioma patients (Bitanihirwe et al., 2014). Dactolisib (BEZ235) treatment inhibited mesothelioma cell growth by targeting mTOR (Zhou et al., 2014) and similarly, treatment with the mTOR inhibitor temsirolimus stopped MPM cell proliferation and was synergistic with cisplatin treatment *in vitro* and *in vivo* (Hoda et al., 2011). It stands to reason that YB-1 overexpression is likely to be, at least in part, linked to the prominent role mTOR signaling plays in thoracic cancers.

### Autoregulation of YB-1 – An Unsolved Piece of the Puzzle

Y-box binding protein-1 is controlled by an autoregulatory feedback loop in which YB-1 binds its own mRNA at two 8 nucleotide motifs at (nt) 1133–1145 and nt 1165–1172, inhibiting translation prior to 40S ribosomal subunit binding (Skabkina et al., 2005; Figure 2). PABP competes with YB-1 at one of these overlapping sites (nt 1149–1196), and stimulates *YBX1* translation (Skabkina et al., 2003, 2005). Considering YB-1 overexpression is frequently observed in cancer, this feedback system may be dysregulated before or during malignant progression. It is possible that PABP upregulation could cause a bias for PABP translational activation of *YBX1*, although PABP itself is controlled by a similar autoregulatory loop (Ma et al., 2006). Nonetheless, recent expression and interactome analysis of YB-1 showed that PABP significantly correlated with YB-1 expression in ADC, implying it plays a central role in YB-1 upregulation and highlighting the need for further investigation into the PABP/YB-1 loop (Murugesan et al., 2018).

### Non-coding RNA Modulate YB-1 Expression

Various families of ncRNAs also play a role in regulating YB-1 levels (Figure 2). One such family are miRNAs – a conserved class of short, ncRNAs that regulate gene expression by binding to and initiating RNA-induced silencing complex-mediated degradation of target mRNA (Jonas and Izaurralde, 2015). The inhibition of *YBX1* by several miRNAs has been shown in other cancers, although to our knowledge such interactions have not been investigated in thoracic cancers. We recently demonstrated regulation of *YBX1* by miR-137 in MPM cells, inhibiting growth, migration and invasion (Johnson et al., 2018). This miRNA is also known to act as a tumor suppressor in lung cancer by targeting SRC3 (Chen R. et al., 2017) and BMP7 (Yang et al., 2015). Another miRNA known to target *YBX1* is miR-216a, which suppresses YB-1-mediated metastasis in pancreatic cancer (Lu et al., 2017). MiR-216a acts as an tumor suppressor in SCLC by targeting and downregulating the anti-apoptotic protein B-cell lymphoma 2 (Bcl-2) (Wang et al., 2018), although it is likely that these effects are also, in part, due to *YBX1* downregulation.

The lncRNA DANCR has been implicated in reducing the levels of this miRNA through its complimentary miR-216a binding site, sequestering it away from miR-216a targets (Zhen et al., 2018). DANCR is associated with advanced tumor grade and poor prognosis in lung cancer and promotes ADC cell growth *in vitro* and *in vivo* (Zhen et al., 2018). Dysregulation of DANCR and subsequent lowering of miR-216a could represent one mechanism of YB-1 overexpression in thoracic cancer, representing an area which requires further investigation.

In addition to DANCR, other lncRNAs as well as transfer RNA-derived fragments can also play a role in regulating YB-1 expression, reviewed previously (Suresh et al., 2018). One example is the lncRNA GAS5, which interacts with YB-1 protein and activates *YBX1* translation, upregulating p21 and initiating G1 cell cycle arrest in stomach cancer (Liu et al., 2015). Interestingly, GAS5 knockdown did not affect *YBX1* mRNA expression, something the authors attribute to possible interactions with other proteins (Liu et al., 2015). GAS5 is a known competing endogenous RNA for miR-137, which targets *YBX1* in thoracic cancers (see above in this section) (Chen et al., 2018), so it is possible that this may contribute toward *YBX1* translational upregulation. However, GAS5 knockdown does not affect *YBX1* mRNA expression (Liu et al., 2015), as would be expected by an increase in miR-137 availability, so this does not fully explain this relationship. Further inquiry into the GAS5/YB-1 and possibly miR-137 relationship is required. GAS5 acts as a tumor suppressor and is lost in lung cancer and mesothelioma (Renganathan et al., 2014; Shi et al., 2015), which is consistent with findings in other cancer types (Gutschner et al., 2018). The apparent discrepancy between the tumor suppressive function of GAS5 and GAS5-mediated translational upregulation of the oncogene *YBX1* remains unanswered and also warrants further study.

More recently, the lncRNA *MIR22HG* was shown to prevent proteasomal degradation of YB-1 in lung cancer cells, which might contribute to YB-1 overexpression (Su et al., 2018). LINC00312 also interacts with YB-1 driving invasion, migration and vascular mimicry of ADC cells, and LINC00312 is associated with metastasis in ADC patients (Peng et al., 2018). HULC is another lncRNA that binds to YB-1 in hepatocellular carcinoma cells, promoting Ser102 phosphorylation, the significance of which is further described in section “Post-Translational Modification in the Control of YB-1 Activity and Localization” (Li et al., 2017). HULC is overexpressed in NSCLC and can promote proliferation via SPHK1 upregulation, which is upstream of the PI3K/AKT pathway (Liu L. et al., 2018). This implies that HULC may also be involved in PI3K-mediated YB-1 activation. TP53TG1, yet another lncRNA, can also bind to YB-1 and inhibit its nuclear translocation, stopping it from transcriptionally activating its oncogenic targets (Diaz-Lagares et al., 2016). TP53TG1 is downregulated in NSCLC and its upregulation sensitized cisplatin resistant NSCLC cells to cisplatin (Xiao et al., 2018). This was attributed to the downregulation of miR-18 (Xiao et al., 2018), however, considering the likely role of YB-1 transcriptional regulation in cisplatin resistance (see section “A Possible Role for YB-1 in Resistance to Platinum-Based Chemotherapy”), it is possible that cytoplasmic retention of YB-1 also played a part in the cisplatin sensitivity seen here. Finally, CAR10 binds to and stabilizes YB-1, leading to the upregulation of EGFR in lung cancer and promoting proliferation (Wei et al., 2016). ncRNA therefore play an integral role in the expression and activity of YB-1, and dysregulation of these families is likely to contribute to YB-1 overexpression in cancer.

### Post-translational Modification in the Control of YB-1 Activity and Localization

**FIGURE 3:**
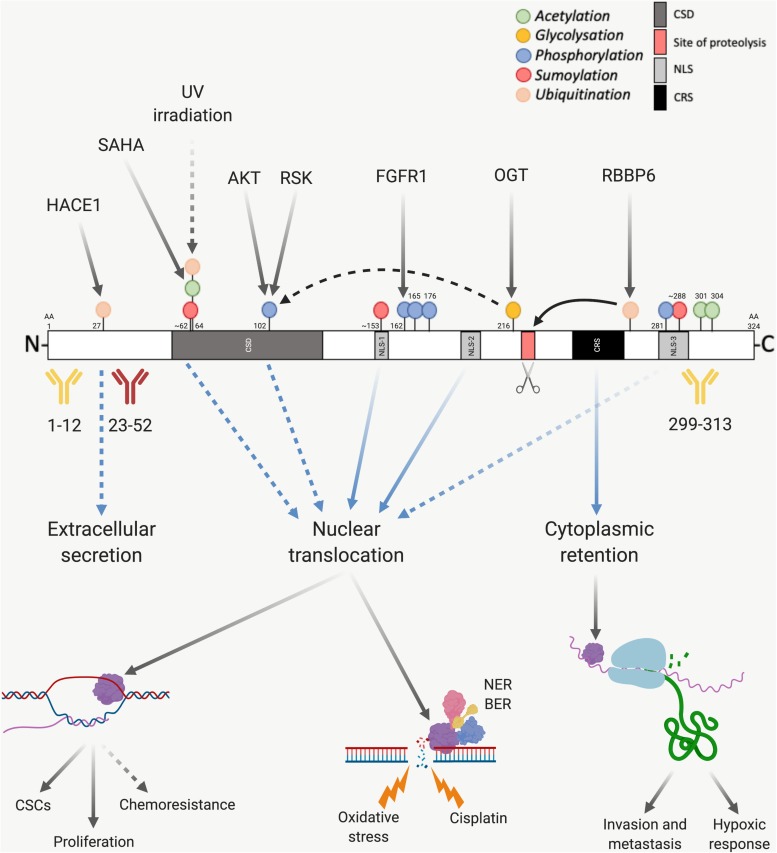
Post-translational modification of YB-1. The presence of various sites before or after proteasomal cleavage of YB-1 modulates its function and localization, which has implications on antibody use. YB-1 is comprised of a CSD shown in dark gray, an N-terminal domain in white and a CTD, also in white. Within the CTD there are three nuclear localization signals (NLS-1 from amino acid (aa) 149–156, NLS-2 from aa 185 to 194 and NLS-3 from aa 276 to 292), shown in light gray, and one cytoplasmic retention signal (CRS from aa 247 to 267), shown in black. YB-1 is proteolytically cleaved at Glu216 and Glu219 (shown in red and highlighted with a scissors icon), which is thought to stimulate YB-1 translocation. Three commonly used antibodies targeting YB-1 are also shown, two of which have been validated using mass spectrometry (in yellow) and one which is known to cross react with hnRNP1A (in red). If the proteolytic theory of YB-1 translocation is correct, this would also have implications on the use of antibodies. Various post-translational modifications also effect the downstream function and nuclear localization of YB-1. Green dots indicate acetylation, yellow glycosylation, blue phosphorylation, red sumoylation and orange ubiquitination. Solid black arrows indicate a post-translational modification that is produced by a known upstream regulator, or a known function of YB-1. Dotted black arrows indicate a post-translational modification or function that is yet to be fully proven. Blue and dotted blue arrows indicate the movement or supposed movement of YB-1 throughout cellular compartments, respectively. Created with BioRender.com.

The activity of YB-1 is modulated through various post-translational modifications (Figure 3), with phosphorylation being the best studied. Ser102 (located in the CSD of YB-1) is currently the most comprehensively studied phosphorylation site. This site is a target of AKT and RSK, making it downstream of both the MAPK/ERK and PI3K/AKT pathways (Sutherland et al., 2005; Stratford et al., 2008; Mendoza et al., 2011). Several additional phosphorylation sites on YB-1 have been identified including Tyr281, which is located within a NLS toward the C-terminal of YB-1 and correlates with the nuclear localization of either a YB-1 C-terminal fragment or full length YB-1 (van Roeyen et al., 2013) (refer to next section for more detail). Tyr162 on YB-1 is also reportedly phosphorylated by FGFR1 (Kasyapa et al., 2009), an important oncogenic driver in lung cancer (Jamal-Hanjani et al., 2017; Friedlaender et al., 2019) and mesothelioma (Schelch et al., 2014; Quispel-Janssen et al., 2018), however, to our knowledge the significance of this modification has not yet been established. Ser165 and Ser176 on YB-1 are also phosphorylated, each promoting distinct groups of nuclear factor-κB target gene expression. This pathway is commonly dysregulated in thoracic cancers and drives cell survival, chemo- and radiotherapy resistance (Chen et al., 2011; Nishikawa et al., 2014; Prabhu et al., 2015; Martin et al., 2017).

In the case of Ser102, it seems that phosphorylation is linked to the hexosamine biosynthetic pathway, in which OGT and O-linked N-acetylglucosamine add or remove N-acetylglucosamine groups to serine or threonine residues, respectively. OGT-mediated O-linked glycosylation of YB-1 at Thr216 aids in the phosphorylation of Ser102 and subsequent transcriptional activity of YB-1 in hepatocellular carcinoma (Liu et al., 2016; Figure 3).

Sumoylation, acetylation and ubiquitination are also prominent post-translational modifications that can contribute toward regulating YB-1 activity and localization. In addition, the nuclear localization of YB-1 has been linked to three NLS, mapped to amino acid residues 149–156 (NLS-1), residues 185–194 (NLS-2) and residues 276–292 (NLS-3) (van Roeyen et al., 2013).

Y-box binding protein-1 is sumoylated at three distinct sites in response to circadian rhythm in zebra fish cells, which has implications on its nuclear shuttling (Pagano et al., 2017). One of these sites is a canonical inverted sumoylation site (at amino acids 287–290 within NLS-3), while the other two are non-canonical sites (at 60–63 which is within the CSD and at 151–154, within NLS-1; Figure 3) (Pagano et al., 2017). Circadian disruption has been correlated with an increased risk of cancer development (Hansen, 2017; Liu W. et al., 2018) and many processes integral to tumorigenesis follow circadian rhythms (cell cycle regulation and DNA repair, for example). Although one study failed to find a link between night shift work and lung cancer among a cohort of female textile workers in Shanghai, China (Kwon et al., 2015), preclinical data indicates that disturbance of the circadian clock can promote lung tumor growth *in vivo* (Papagiannakopoulos et al., 2016). Modulation of YB-1 localization in response to light may represent one contributing factor in the observed correlation between circadian rhythm and cancer and warrants further investigation.

Acetylation of YB-1 has been reported to occur in lung cancer cells, however, the significance of this remains unclear. YB-1 was one of 542 proteins acetylated by the histone deacetylase inhibitor SAHA in SILAC experiments in a NSCLC cell line (Wu et al., 2015). Here, YB-1 was acetylated at Lys64 (Figure 3). Lys301/304 of YB-1 can also be acetylated and the amount of acetylated YB-1 is significantly increased in monocytes of hemodialysis patients (Ewert et al., 2018).

**FIGURE 4:**
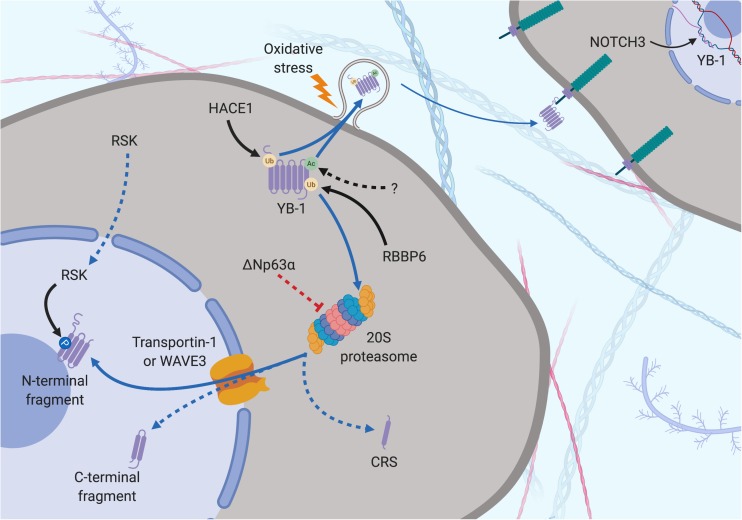
Subcellular localization of YB-1 – the proteolytic theory of nuclear localization. YB-1 can be found in the nucleus, cytoplasm and extracellular space and its localization is mediated by various factors. Secretion can be preceded by Ubiquitination (orange dot) by HACE1 and acetylation (green dot) by a currently unknown protein. Oxidative stress stimulates stress-granule localization and eventual section of YB-1, where it can bind to the transmembrane protein Notch3 on other cells. YB-1 is cleaved by the proteasome prior to nuclear translocation. Ubiquitination by RBBP6 initiates YB-1 proteolytic cleavage. ΔNp63α prevents full length proteolysis by partially inhibiting YB-1 degradation, resulting in the removal of the CRS. Transportin-1 or WAVE3 bind to NLS of YB-1 and translocate it to the nucleus. RSK can cross into the nucleus, phosphorylating nuclear YB-1 fragments. Solid black arrows indicate a post-translational modification that is produced by a described or known mechanism. Dotted black arrows indicate a post-translational modification whose significance is yet to be realized. Blue and dotted blue arrows indicate the movement or supposed movement of YB-1 throughout cellular compartments, respectively. Created with BioRender.com.

Ubiquitination may also play an important role in YB-1 expression and subcellular localization. RBBP6 initiates proteasomal degradation of YB-1 by binding to and ubiquitinating it within a 62-residue fragment of the YB-1 CTD (Chibi et al., 2008). The protein isoform of p63 ΔNp63α counteracts this by preventing proteolysis of full-length YB-1 and stimulating accumulation of poly-ubiquitinated YB-1 in the nucleus (di Martino et al., 2016), possibly supporting the role of proteolytic cleavage-dependent YB-1 nuclear shuttling (discussed further in section “Control of YB-1 Subcellular Localization”; Figures 3, 4). Further supporting this theory, UV irradiated DNA damage stimulates YB-1 ubiquitination at Lys64 (Boeing et al., 2016) (the same lysine residue that is acetylated, above in this section; Figure 3). Considering the DNA repair function of YB-1 and the aforementioned ubiquitination-driven proteasomal cleavage of YB-1, this possibly induces a similar nuclear translocation of YB-1. This is further supported by results showing that YB-1 is shuttled to the nucleus upon UV irradiation (Koike et al., 1997).

Ubiquitination is also important in the secretion of YB-1 via the multi-vesicular body pathway. The E3 ligase activity of HACE1 polyubiquitinates YB-1 at K27, facilitating tumor susceptibility gene 101 binding, which initiates YB-1 secretion (Palicharla and Maddika, 2015). In summary, post-translational modification influences the levels, activity and localization of YB-1, which in turn impacts the downstream effects of YB-1.

### Control of YB-1 Subcellular Localization

In non-malignant cells, YB-1 is primarily located in the cytoplasm and functions as a major component of free messenger ribonucleoprotein complexes, where it can inhibit or stimulate cap-dependent translation depending on the ratio of YB-1 to mRNA (Suresh et al., 2018). Under certain stresses such as cisplatin treatment (Yahata et al., 2002), hypoxia (Rauen et al., 2016), UV radiation (Koike et al., 1997), and hyperthermia (Stein et al., 2001), YB-1 translocates to the nucleus, however, the underlying mechanism of this remains unclear. As above, YB-1 has three NLS sites which have been mapped to amino acid residues 149–156, residues 185–194 and residues 276–292 (van Roeyen et al., 2013), which are recognized by transportin-1 (Mordovkina et al., 2016) and WAVE3 (Bledzka et al., 2017). In addition YB-1 also contains a CRS at residues 247–267 (Woolley et al., 2011; Figure 3). The locations of these sites are postulated to regulate YB-1 nuclear-cytoplasmic translocation.

One line of evidence suggests that nuclear translocation is preceded by a specific proteolytic cleavage by the 20S proteasome of YB-1 at Glu216 and Glu219 under cellular stress (Sorokin et al., 2005; Kim et al., 2013; Figures 3, 4). This results in loss of a 105-amino acid sequence from the C-terminus, including the CRS, and accumulation of the remaining N–terminal fragment in the nucleus (Sorokin et al., 2005; Kim et al., 2013). The presence of an NLS in the CTD suggests that a C–terminal fragment may also be shuttled to the nucleus, presumably if the nearby CRS has been cleaved off (van Roeyen et al., 2013; Figures 3, 4). Supporting this, breast cancer cells preincubated with the proteasome inhibitor MG-132 before treatment with doxorubicin displayed reduced nuclear and enhanced cytoplasmic levels of YB-1 (visualized with a C-terminal-targeting antibody; Figure 3), compared to cells treated with doxorubicin alone (van Roeyen et al., 2013). However, this does not rule out whether full-length YB-1 translocation occurs by some other mechanism.

**FIGURE 5:**
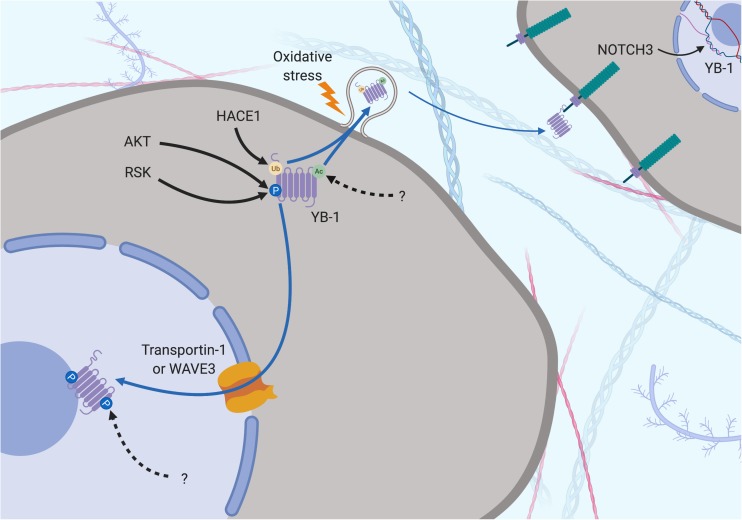
Subcellular localization of YB-1 – the phosphorylation theory of nuclear localization. YB-1 can be found in the nucleus, cytoplasm and extracellular space and its localization is mediated by various factors. Secretion can be preceded by Ubiquitination (orange dot) by HACE1 and acetylation (green dot) by a currently unknown protein. Oxidative stress stimulates stress-granule localization and eventual section of YB-1, where it can bind to the transmembrane protein Notch3 on other cells. Phosphorylation is required before nuclear shuttling can take place. Ser102 is phosphorylated by upstream kinases, changing the configuration of YB-1 to block the CRS and allow nuclear shuttling via Transportin-1 or WAVE3. Phosphorylation of Tyr281 by a currently unknown upstream regulator may play a role here too. Solid black arrows indicate a post-translational modification that is produced by a described or known mechanism. Dotted black arrows indicate a post-translational modification whose significance is yet to be realized. Blue and dotted blue arrows indicate the movement or supposed movement of YB-1 throughout cellular compartments, respectively. Created with BioRender.com.

Countering the proteasomal theory is one study that suggests the YB-1 N-terminal fragment has been misidentified as another protein, hnRNP1A, and that only full-length YB-1 is found in the nucleus (Cohen et al., 2010). Full-length YB-1 nuclear translocation could be facilitated by its phosphorylation. For example, there is evidence showing that YB-1 is phosphorylated at Ser102 by the serine/threonine kinase AKT before being shuttled to the nucleus (Sutherland et al., 2005; Figures 3, 5). This may cause a conformational change which could block the CRS of YB-1, stimulating its nuclear shuttling. However, a recent study found that while ionizing radiation, EGF stimulation and overexpression of the KRAS G12V mutant induced Ser102 phosphorylation of YB-1 in both the nucleus and the cytoplasm, there was no increase in YB-1 expression in nuclear fractions (Tiwari et al., 2018). The authors attribute this to nuclear translocation of RSK, phosphorylating pre-existing nuclear YB-1 – not the shuttling of YB-1 itself (Figure 4). It may be that the translocation of either YB-1, RSK or both is dependent on the type of cellular stress applied. As mentioned in section “Post-Translational Modification in the Control of YB-1 Activity and Localization,” phosphorylation of Thr281 within the NLS 276–292 of YB-1 also correlates with its nuclear translocation (van Roeyen et al., 2013), however, it is not yet clear whether this modification is actively involved in YB-1 shuttling.

The localization of YB-1 also appears to be dependent on its ability to bind RNA and other proteins in the cytoplasm as YB-1 nuclear localization is hampered by higher cytoplasmic mRNA levels (Tanaka et al., 2018). This group also found that p53 (along with 4 other nucleocytoplasmic-shuttling proteins) binds to a YB-1 NLS and co-accumulates with YB-1 in the nucleus in response to actinomycin D treatment (Tanaka et al., 2016). This implies that YB-1 nuclear localization is a p53-mediated response to DNA stress. Another factor, C1QBP inhibits nuclear localization by binding to and blocking an NLS region (Matsumoto et al., 2018). C1QBP binding also moderately attenuated YB-1-mediated mRNA stabilization (Matsumoto et al., 2018). It is likely that the balance of this cytoplasmic interactome determines where YB-1 is localized under different conditions and that a disruption of this balance may lead to malignant progression.

### YB-1 Is Secreted Into the Extracellular Space Under Cellular Stress

Stress-related secretion of factors found in the nucleus and cytoplasm have been found to be biologically relevant in thoracic cancer biology and may serve as potential non-invasive biomarkers. Secretion of the nuclear protein HMGB1 in response to asbestos-related necrosis in MPM cells, which acts as an alarmin to stimulate inflammation is one example (Yang et al., 2010). Serum HMGB1 has shown prognostic relevance as a possible biomarker in MPM (Tabata et al., 2013).

Y-box binding protein-1 is related on an evolutionary level to HMGB1 and is also secreted under certain cellular stresses. This was first evident in monocytes stimulated with bacterial lipopolysaccharide through an active, non-classical pathway and appears to require the same two lysine residues (Lys301/304) that are the site of acetylation in hemodialysis patients (Frye et al., 2009; Ewert et al., 2018; Figures 3–5). Secreted YB-1 stimulated DNA synthesis, cell proliferation and migration of kidney cells (Frye et al., 2009). More pertinent to thoracic cancer, YB-1 is also secreted under oxidative stress. YB-1 translationally upregulates *G3BP1* under oxidative stress and localizes to cytoplasmic stress granules where it is involved in pro-survival mRNA reprogramming (Somasekharan et al., 2015). G3BP1 also promotes the invasion and metastasis of sarcoma cells *in vivo* (Somasekharan et al., 2015). In support, YB-1 enrichment in stress granules is also linked to its secretion to the extracellular space under oxidizing conditions (Guarino et al., 2018; Figures 4, 5). Secretion of YB-1 resulted in depletion of cytoplasmic YB-1, leaving nuclear expression intact (presumably to allow for YB-1-mediated DNA repair), while secreted YB-1 inhibited the growth of neighboring keratinocytes (Guarino et al., 2018).

Extracellular YB-1 acts as a ligand for Notch3, binding to epidermal growth factor-like repeats 17–24 on Notch3 and subsequently promoting *YBX1* translation in a feed-forward, autoregulatory fashion (Rauen et al., 2009; Raffetseder et al., 2011; Gera and Dighe, 2018; Figures 4, 5). Notch3 is frequently overexpressed in NSCLC where high levels correlate with poor prognosis, making it a candidate target for therapeutic intervention (Zong et al., 2016). Considering the prevalence of oxidative stress and Notch3 in thoracic cancers, the secretion of YB-1 may be biologically important, although more studies are required to fully understand this process.

## YB-1 in Thoracic Cancers: Clinical Relevance

### YB-1 as a Prognostic Biomarker

There is evidence supporting the use of YB-1 as a prognostic biomarker in thoracic cancers, and subcellular localization is important in this regard. Analysis of TCGA data demonstrate that high levels of *YBX1* mRNA correlate significantly with poor prognosis in NSCLC and MPM patients (Figures 1D,E). YB-1 protein has been correlated with poor prognosis previously (Shibahara et al., 2001; Gessner et al., 2004), supported by a recent meta-analysis of six studies covering data on 692 NSCLC patients, where YB-1 was associated with worse overall survival, tumor stage and depth of invasion (Jiang et al., 2017). A study in MPM patients also supports the TCGA data (albeit tentatively due to the low number of patients in the cohort) (Iwanami et al., 2014). Here, YB-1 levels were shown to be higher in sarcomatoid MPM tumors, which confer the worst prognosis (Iwanami et al., 2014).

There has been some contention surrounding the use of particular YB-1 antibodies in prognostic studies across cancer types. One N-terminal targeting YB-1 antibody that binds to residues 23–52 has been used in prognostic studies in the past (Figure 3). However, this antibody has been shown to cross reacts with the ubiquitously expressed hnRNPA1 protein via mass spectrometry making it unsuitable for such application (Woolley et al., 2011). Antibodies targeting the extreme N-‘terminus of YB-1 (residues 1–12) or residues 299–313 in the CTD (C-terminal) have been shown to be specific for YB-1, again by mass spectrometry (Woolley et al., 2011; Figure 3). However, the N–terminal antibody has been suggested as more suitable for prognostic applications as this region does not interact with other proteins, so this epitope may be more accessible (Woolley et al., 2011). Notably, all prognostic studies cited in this review utilize the C-terminal targeting antibody. Regardless, a universal standardization of one reliable antibody would significantly enhance the prognostic potential of YB-1 for diagnosis using traditional pathological tissue staining.

Secreted YB-1 may also have prognostic significance in cancer. One study of 44 breast cancer patients with bone metastases found that serum YB-1 was present in 50% of patients and associated with extra-bone metastases and faster bone disease progression (Ferreira et al., 2017). There was a trend toward poorer overall survival in high-YB-1 patients, although a bigger cohort is needed to provide a more definitive answer (Ferreira et al., 2017). Another group found an YB-1/p18 in the plasma of patients with various diseases (including 32/38 lung cancers) but not in healthy controls via Western Blot using a monoclonal YB-1 antibody (Tacke et al., 2014). This study found no prognostic significance of YB-1/p18 in any of the cancers tested, but they assert that YB-1/p18 may have diagnostic significance (Tacke et al., 2014). The small sample number in this study should be noted before the prognostic applicability of secreted YB-1 is ruled out. Investigating the prognostic significance of secreted full-length or other fragments of YB-1, not just YB-1/p18, may also be of interest. The potential of YB-1 as a circulating biomarker is intriguing as a non-invasive method of prognosis and diagnosis, although more studies with larger cohorts are required.

### Targeting YB-1: An Achievable Feat?

In the past YB-1 has been overlooked as a therapeutic target because of its role as a transcription and translation factor, which have been traditionally hard to target with small molecule inhibitors. However, recent advancements in the delivery of RNA-based drugs has opened up new potential avenues of targeting oncoproteins such as YB-1 (Seton-Rogers, 2012; Afonin et al., 2014). We and others have shown that miRNA or siRNA can be used to target *YBX1* in thoracic cancer cells in preclinical studies (Xu et al., 2015; Johnson et al., 2018). The delivery of miRNA mimics in the clinic is now thought to be a viable anti-cancer strategy. For example, MRX34 (a liposomal miR-34a mimic) showed evidence of efficacy and safety in a phase I trial in patients with various solid tumors including 1 NSCLC patient (Beg et al., 2017). More pertinently, a phase 1 clinical trial delivering miR-16-based mimics using bacterial minicells (EnGeneIC Dream Vectors) in mesothelioma and advanced NSCLC patients demonstrated the safety and efficacy of miRNA-based therapy (van Zandwijk et al., 2017), evidencing the potential for miRNA replacement therapy in patients with thoracic cancer.

There are a number of systems which pose as attractive options to deliver RNA-based drug payloads in thoracic cancer such as lipid, RNA, inorganic and polymer-based nanoparticles, all with their respective advantages and drawbacks (Shu et al., 2014). The delivery of siRNA or miRNA using nanoparticles in lung cancers, and to a lesser extent MPM, has been achieved *in vitro* and *in vivo*, evidencing the potential of these delivery systems (Lee et al., 2016). The *in vivo* transport of siRNA to large cell lung carcinoma tumors using lipoprotein nanoparticles (Tagalakis et al., 2018) and ADC tumors using polyethylene glycol nanoparticles (Wen et al., 2017) has demonstrated the applicability of nanoparticle delivery systems for targeted therapy. However, these studies treated subcutaneously grown tumors, which do not reflect the orthotopic context of thoracic cancer and the problems with delivery that come with it. Recently though, an siRNA targeting anti-*EZH2* was successfully delivered to orthotopically grown NSCLC tumors in mice using modified polyethyleneimine nanoparticles (Yuan et al., 2017), and delivery and retention of amiloride-sensitive epithelial sodium channel-specific siRNA into the lungs of normal mice was achieved (Tagalakis et al., 2018). The successful delivery of miR-215-5p miRNA mimics complexed in an atelocollagen vehicle was also recently achieved in an orthotopic MPM mouse model, which significantly suppressed tumor growth (Singh et al., 2019). The advances in RNA-based drug delivery in preclinical and clinical studies mean that siRNA or miRNA delivery is an appealing YB-1 targeting strategy in thoracic cancers. However, other potential strategies may also be of interest, although these are yet to be investigated in humans.

Inhibiting YB-1 activation may be one such viable targeting strategy. Fisetin (3,7,3′,4′-tetrahydroxy flavone) is a flavanol that binds to the CSD of YB-1, inhibiting its phosphorylation at Ser102 and blocking EMT in prostate cancer cells *in vitro* (Khan et al., 2014). Targeting YB-1 using fisetin also attenuated the growth of melanoma cells *in vitro* and *in vivo* (Sechi et al., 2018). Fisetin was also found to inhibit mTOR and PI3K/Akt signaling in NSCLC cells, both of which are important in both thoracic cancer biology and YB-1 regulation (see section “Translational Regulation of YB-1”) (Khan et al., 2012). Another possible method for targeting YB-1 was demonstrated by using an interference cell permeable peptide that prevented YB-1 Ser102 phosphorylation. This led to an inhibition of EGFR expression and reduced growth of prostate and breast cancer cells, but not of non-malignant mammary epithelial cells (Law et al., 2010). Upstream inhibitors such as those targeting mTOR may also be an option (Hoda et al., 2011; Zhou et al., 2014), but would not be specific. A recent study showed that 2,4-dihydroxy-5-pyrimidinyl imidothiocarbomate antagonizes YB-1, inhibits YB-1 nuclear translocation and increases doxorubicin accumulation in breast cancer cells (Gunasekaran et al., 2018).

The use of oncolytic viruses that require YB-1 for replication is another potential therapeutic approach. XVir-N-31-mediated lysis of brain CSCs and virus production was significantly reduced in non-malignant astrocyte cells that expressed significantly less YB-1 compared to CSC cells (Mantwill et al., 2013). XVir-N-31 also repressed the growth of bladder cancer cells with strong YB-1 expression *in vitro* and intra-tumor delivery significantly repressed tumor volume *in vivo* (Lichtenegger et al., 2018). Consequently, virotherapy may prove to be an interesting avenue for targeting YB-1 overexpressing lung cancer and MPM.

Preclinical evidence in other tumors suggests that targeting YB-1 could also benefit immunotherapy in some cases. YB-1 knockdown increased the efficacy of IFN-α in renal cell carcinoma cells *in vitro* and *in vivo* (Takeuchi et al., 2013). IFN-α in combination with cisplatin provided a partial response in five out of ten patients in an open-label non-comparative phase II study of NSCLC patients (Chao et al., 1995). A phase II randomized study in SCLC patients with limited disease also showed a survival benefit of IFN-α in combination with a chemotherapy regime of carboplatin, ifosfamide and etoposide (Zarogoulidis et al., 2013). Based on these results, further investigating whether targeting YB-1 could increase the modest efficacy of IFN-α in thoracic cancer is warranted.

The use of YB-1 as a tumor-associated antigen in therapeutic vaccination has also shown promise in other cancers. YB-1 was identified as a tumor-associated antigen in neuroblastoma by serological expression of cDNA expression libraries (Zheng et al., 2009). In the context of regulatory T-cell depletion, YB-1 immunization enhanced CD8^+^ response against neuroblastoma cells and conferred significantly higher mouse survival compared to control groups (Zheng et al., 2012). Adoptive T-cell therapy from immunized mice into neuroblastoma tumor-bearing mice also conferred a significant survival benefit and reduced tumor growth (Zheng et al., 2012). Again, further study in the context of thoracic cancer is warranted.

It must be noted that as with all current targeted therapies, it is likely that a YB-1-based approach to thoracic cancer management would benefit only a sub-population of patients. YB-1 overexpression, rather than mutation, would probably be the best predictive marker as mutations of YB-1 are very rare (Cerami et al., 2012; Gao et al., 2013). TCGA data (Figure 1) suggests that ∼10% of thoracic cancer patients would benefit, making it comparable to ALK inhibitors in ADC according to these datasets.

## Future Directions and Areas Requiring Further Study

Throughout this review we have highlighted some avenues for potential future research that currently require further consideration, summarized briefly below. The YB-1/SOX2 axis needs to be further investigated in lung cancer, particularly in SCC and SCLC where the development of new therapeutic strategies is most urgent. The feed-forward loop of YB-1 and Myc also requires further investigation in the context of thoracic cancer. The roles of certain ncRNA in the dysregulation of YB-1 are also still unclear, namely the relationship between GAS5, miR-137 and YB-1 and the potential DANCR/miR-216a/YB-1 loop. Also, the apparent tumor suppressor function of GAS5 does not fit with its role in promoting YB-1 translation, which is another area requiring further attention.

While there is strong evidence supporting YB-1-driven resistance to platinum chemotherapy in other cancers, a study looking at the effect of YB-1 knockdown on cisplatin or other platinum drug sensitivity in lung cancer or MPM cells is still required. Also, while YB-1 has been shown to upregulate LRP and MRP1, the effect of these interactions on cisplatin resistance are yet to be determined. Determining the involvement of YB-1 in thoracic cancer exosomes would also be of interest. And while the mechanism underlying YB-1-driven growth in lung cancer has been studied well, similar studies in MPM cells are yet to be conducted.

Perhaps the most contentious area warranting further study relates to the regulation of YB-1 localization. Determining whether the proteolytic theory, phosphorylation theory or both is correct remains an important determination to be made. While these theories represent the most studied lines of evidence covering YB-1 nuclear localization, other post-translational modifications could also play a role and deserve further attention, including the phosphorylation of Tyr281. However, what upstream regulator phosphorylates YB-1 here and whether this post-translational modification actually plays an important role is not yet known. Determining whether sumoylation and circadian-related YB-1 translocation occurs and is important in lung cancer and MPM patients would also be of interest.

The secretion of YB-1 into the extracellular space in response to oxidative stress has been reported in other cell types but is yet to be studied in thoracic cancers. If secretion does occur in these contexts, it would be interesting to determine whether acetylation of Lys301/304 is required, as in immune cells. Evaluating the potential interaction between secreted YB-1 and Notch3 here would also be interesting. It is also possible that secreted YB-1 could be used as a biomarker down the line, however, studies with larger patient numbers are required to determine this. Regardless, the evidence supports utilizing YB-1 as a prognostic tissue biomarker, however, universal standardization of an appropriate YB-1 antibody is would be favorable.

**FIGURE 6:**
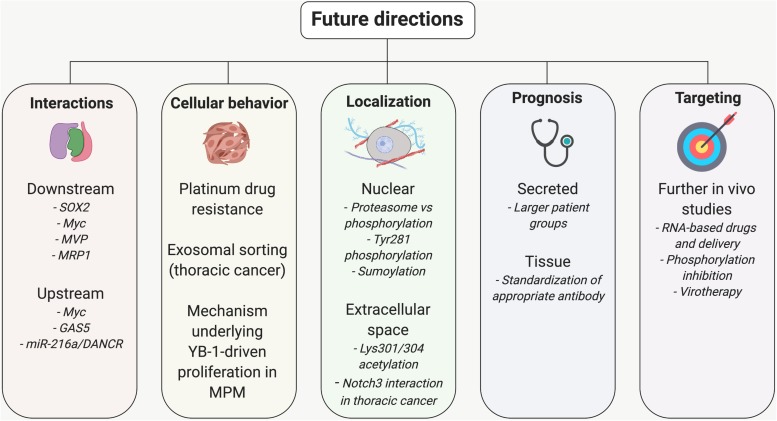
Further study required to understand the role of YB-1 and use it in the treatment and management of thoracic cancer patients. Various upstream and downstream regulatory loops and the role of YB-1 in platinum drug resistance, exosomal sorting and proliferation need further study to fully understand the biology of YB-1 in lung cancer and MPM. The mechanism of YB-1 nuclear localization is also under contention and the occurrence and significance of secreted YB-1 is yet to be determined. Standardization of a suitable YB-1 antibody for prognostic application would also be a step forward. Finally, evaluating the current strategies of YB-1 inhibition *in vivo* further would build a stronger case for translation into humans. Created with BioRender.com.

Finally, YB-1 remains an interesting target in thoracic cancer, but further *in vivo* studies delivering YB-1-targeting drugs need to be done before translation into humans can occur. This section is summarized in Figure 6.

## Conclusion

In summary, this review covers recent advances in the understanding of YB-1 in cancer biology with a focus on thoracic cancers. YB-1 plays an important role in the malignant behaviors of lung cancer and MPM including proliferation, invasion and metastasis. It also has been shown to be involved in the maintenance and behavioral regulation of CSCs. The demonstrated prognostic significance of YB-1 and developments in the delivery of RNA-based drugs mean that utilizing this multifunctional oncoprotein in the management of thoracic cancer may soon become a reality.

## Author Contributions

This review was drafted by TJ and critically revised by KS, SM, AB, and GR.

## Conflict of Interest

GR has an issued patent, US 9,006,200, covering use of microRNAs for cancer therapy. The remaining authors declare that the research was conducted in the absence of any commercial or financial relationships that could be construed as a potential conflict of interest.

## Abbreviations

ΔNP63α: Isoform p63α – protein isoform of p63
ADC: lung adenocarcinoma
AKT: lrotein kinase B
ALK: anaplastic lymphoma kinase
APE1: apurinic/apyrimidinic endonuclease 1
BAP1: BRCA associated protein-1
Bcl-2: B-cell lymphoma 2
BER: base-excision repair
BMP7: bone morphogenetic protein 7
BRAF: proto-oncogene B-Raf
C1QBP: complement component 1 Q subcomponent-binding protein
CAR10: CAR intergenic 10
CCND1: cyclin D1
CDC25A: M-phase inducer phosphatase 1
CDKN2A: cyclin-dependent kinase inhibitor 2A
COPD: chronic obstructive pulmonary disease
CRS: cytoplasmic retention signal
CSC: cancer stem-like cell
CSD: cold shock domain
CTD: C-terminal domain
DANCR: differentiation antagonizing non-protein coding RNA
E/M: hybrid epithelial/mesenchymal state
EGF: epidermal growth factor
EGFR: epidermal growth factor receptor
EMT: epithelial-mesenchymal transition
EPO: human erythropoietin
EZH2: enhancer of zeste homolog 2
FGFR1: fibroblast growth factor receptor 1
FOXO3a: forkhead box O3
G3BP1: Ras GTPase-activating protein-binding protein-1
GAS5: growth arrest specific 5
HACE1: HECT domain and ankyrin repeat containing E3 ubiquitin protein ligase 1
HER2: human epidermal growth factor receptor 2
HIF1 α: Hypoxia-inducible factor 1- α
HMGB1: high mobility group box 1 protein
HULC: highly up-regulated in liver cancer
LGC: large cell carcinoma
LINC00312: long intergenic non-protein coding RNA 312
LMO3: LIM domain only protein 3
lncRNA: long non-coding RNA
LRP/MVP: lung resistance protein/major vault protein
MAPK/ERK: Mitogen-activated protein kinase/extracellular-signal-regulated kinase
MDCK: Madin-Darby canine kidney cell
MDR1: multi-drug resistance 1
MET: tyrosine-protein kinase Met
MIR22HG: MIR22 host gene
miRNA: microRNA
MPM: malignant pleural mesothelioma
MRP1: multidrug resistance-associated protein 1
mTOR: mammalian target of rapamycin
NANOG: homeobox protein NANOG
ncRNA: non-coding RNA
NEIL1: nei like DNA glycosylase 1
NER: nuclear excision repair
NF2: neurofibromatosis type 2
NLS: nuclear localization signal
Notch1/*NOTCH1*: notch homolog 1
Notch3: notch receptor 3
NSCLC: non-small cell lung cancer
nt: nucleotide
Oct4: octamer-binding transcription factor 4
OGT: O-linkedN-acetylglucosamine transferase
PABP: Poly(A)-binding protein
PARP1: Poly(ADP-ribose) polymerase 1
PARP2: Poly(ADP-ribose) polymerase 2
PCNA: proliferating cell nuclear antigen
PIK3CA: phosphatidylinositol-4,5-bisphosphate 3-kinase, catalytic subunit α
RBBP6: retinoblastoma binding protein 6
RSK p90: ribosomal S6 kinase
SAHA: suberoylanilide hydroxamic acid
SCC: lung squamous carcinoma
SCLC: small cell lung cancer
SETD2: SET domain containing 2
SILAC: stable isotope labeling with amino acids in cell culture
siRNA: small interfering RNA
Snail: Zinc finger protein SNAI1
SOX2: sex determining region Y-box 2
SPHK1: sphingosine kinase 1
SRC3: steroid receptor co-activator 3
ssDNA: single-stranded DNA
TCGA: The Cancer Genome Atlas
TGF β 1: transforming growth factor β 1
*TP53*/p53: tumor protein 53
TP53TG1: TP53 target 1
Twist1: Twist-related protein 1
XPC: xeroderma pigmentosum
YB-1: Y-box binding protein-1
YB-1/p18: 18 kDa fragment of YB-1.
