# Corrigendum: Why Be One Protein When You Can Affect Many? The Multiple Roles of YB-1 in Lung Cancer and Mesothelioma

**DOI:** 10.3389/fcell.2019.00293

**Published:** 2019-11-22

**Authors:** Thomas G. Johnson, Karin Schelch, Sunali Mehta, Andrew Burgess, Glen Reid

**Affiliations:** ^1^Asbestos Diseases Research Institute, Sydney, NSW, Australia; ^2^Cell Division Laboratory, The ANZAC Research Institute, Sydney, NSW, Australia; ^3^School of Medicine, The University of Sydney, Sydney, NSW, Australia; ^4^Sydney Catalyst Translational Cancer Research Centre, The University of Sydney, Sydney, NSW, Australia; ^5^Institute of Cancer Research, Medical University of Vienna, Vienna, Austria; ^6^Department of Pathology, University of Otago, Dunedin, New Zealand; ^7^Maurice Wilkins Centre, University of Otago, Dunedin, New Zealand

**Keywords:** lung cancer, mesothelioma, targeted therapy, biomarker, Y-box binding protein-1

In the original article, there was an error. The authors wrote “adenylation” instead of “acetylation”.

A correction has been made to the **YB-1: a malignant jack of all trades** section, subsection **YB-1 is secreted into the extracellular space under cellular stress**, paragraph two:

“Y-box binding protein-1 is related on an evolutionary level to HMGB1 and is also secreted under certain cellular stresses. This was first evident in monocytes stimulated with bacterial lipopolysaccharide through an active, non-classical pathway and appears to require the same two lysine residues (Lys301/304) that are the site of acetylation in hemodialysis patients (Frye et al., 2009; Ewert et al., 2018; Figures 3–5). Secreted YB-1 stimulated DNA synthesis, cell proliferation and migration of kidney cells (Frye et al., 2009). More pertinent to thoracic cancer, YB-1 is also secreted under oxidative stress. YB-1 translationally upregulates *G3BP1* under oxidative stress and localizes to cytoplasmic stress granules where it is involved in pro-survival mRNA reprogramming (Somasekharan et al., 2015). G3BP1 also promotes the invasion and metastasis of sarcoma cells *in vivo* (Somasekharan et al., 2015). In support, YB-1 enrichment in stress granules is also linked to its secretion to the extracellular space under oxidizing conditions (Guarino et al., 2018; Figures 4, 5). Secretion of YB-1 resulted in depletion of cytoplasmic YB-1, leaving nuclear expression intact (presumably to allow for YB-1-mediated DNA repair), while secreted YB-1 inhibited the growth of neighboring keratinocytes (Guarino et al., 2018).”

The authors apologize for this error and state that this does not change the scientific conclusions of the article in any way. The original article has been updated.
